# Automated Electronic Frailty Index–Identified Frailty Status and Associated Postsurgical Adverse Events

**DOI:** 10.1001/jamanetworkopen.2023.41915

**Published:** 2023-11-06

**Authors:** Ashish K. Khanna, Vida Motamedi, Bethany Bouldin, Timothy Harwood, Nicholas M. Pajewski, Amit K. Saha, Scott Segal

**Affiliations:** 1 Department of Anesthesiology, Wake Forest University School of Medicine, Winston-Salem, North Carolina; 2 Perioperative Outcomes and Informatics Collaborative (POIC), Winston-Salem, North Carolina; 3 Outcomes Research Consortium, Cleveland, Ohio; 4 Department of Anesthesiology, Vanderbilt University Medical Center, Nashville, Tennessee; 5 Department of Biostatistics and Data Science, Wake Forest University School of Medicine, Winston-Salem, North Carolina

## Abstract

**Importance:**

Electronic frailty index (eFI) is an automated electronic health record (EHR)–based tool that uses a combination of clinical encounters, diagnosis codes, laboratory workups, medications, and Medicare annual wellness visit data as markers of frailty status. The association of eFI with postanesthesia adverse outcomes has not been evaluated.

**Objective:**

To examine the association of frailty, calculated as eFI at the time of the surgical procedure and categorized as fit, prefrail, or frail, with adverse events after elective noncardiac surgery.

**Design, Setting, and Participants:**

This cohort study was conducted at a tertiary care academic medical center in Winston-Salem, North Carolina. The cohort included patients 55 years or older who underwent noncardiac surgery of at least 1 hour in duration between October 1, 2017, and June 30, 2021.

**Exposure:**

Frailty calculated by the eFI tool. Preoperative eFI scores were calculated based on available data 1 day prior to the procedure and categorized as fit (eFI score: ≤0.10), prefrail (eFI score: >0.10 to ≤0.21), or frail (eFI score: >0.21).

**Main Outcomes and Measures:**

The primary outcome was a composite of the following 8 adverse component events: 90-item Patient Safety Indicators (PSI 90) score, hospital-acquired conditions, in-hospital mortality, 30-day mortality, 30-day readmission, 30-day emergency department visit after surgery, transfer to a skilled nursing facility after surgery, or unexpected intensive care unit admission after surgery. Secondary outcomes were each of the component events of the composite.

**Results:**

Of the 33 449 patients (median [IQR] age, 67 [61-74] years; 17 618 females [52.7%]) included, 11 563 (34.6%) were classified as fit, 15 928 (47.6%) as prefrail, and 5958 (17.8%) as frail. Using logistic regression models that were adjusted for age, sex, race and ethnicity, and comorbidity burden, patients with prefrail (odds ratio [OR], 1.24; 95% CI, 1.18-1.30; *P* < .001) and frail (OR, 1.71; 95% CI, 1.58-1.82; *P* < .001) statuses were more likely to experience postoperative adverse events compared with patients with a fit status. Subsequent adjustment for all other potential confounders or covariates did not alter this association. For every increase in eFI of 0.03 units, the odds of a composite of postoperative adverse events increased by 1.06 (95% CI, 1.03-1.13; *P* < .001).

**Conclusions and Relevance:**

This cohort study found that frailty, as measured by an automatically calculated index integrated within the EHR, was associated with increased risk of adverse events after noncardiac surgery. Deployment of eFI tools may support screening and possible risk modification, especially in patients who undergo high-risk surgery.

## Key Points

**Question:**

What is the association between frailty status, as defined by a validated automated electronic frailty index (eFI), and postoperative adverse events in patients recovering from major noncardiac surgery?

**Findings:**

In this cohort study of 33 449 patients who underwent noncardiac surgery lasting at least 1 hour, patients who were classified as prefrail and frail vs those with fit status had a higher odds of postoperative adverse events in a fully adjusted model. Each increase of the eFI score by 0.03 units increased the odds of postoperative adverse events.

**Meaning:**

Findings of this study suggest that frailty status is associated with increased odds of postoperative adverse events in older adults recovering from noncardiac surgery; deployment of eFI tools could support screening and possible risk modification, especially in this population.

## Teaser

This cohort study evaluates the use of a frailty index to estimate risk of postoperative adverse outcomes in patients 55 years or older who are recovering from noncardiac surgery.

## Introduction

Frailty is variably described as a quantifier of a patient’s physiologic reserve at the time of evaluation, a set of findings on clinical examination, or an accumulation of a patient’s health-related deficits.^1,2,3^ Increased frailty is known to be associated with poor outcomes in older adults^4^; however, reliably quantifying frailty as a consistent and easily obtainable metric has remained an elusive task due to factors such as lack of uniformity in comorbidity reporting.^5,6,7^ Objective rating scales for frailty often require considerable time and resources to assess and implement. In addition to these resource constraints, in-clinic assessments of physiologic reserve, which often include patient questionnaires and physical examinations, entail subjective factors, such as patient self-report and interclinician variability, that may contribute to misclassification and bias.

The deficit accumulation model of frailty posits that a higher prevalence of aging-related health deficits is associated with increased frailty risk. An automated approach that captures such deficits has been developed in the form of the electronic frailty index (eFI), a scalable frailty screening instrument for a range of adverse outcomes in both primary care^2^ and surgical settings.^1,3^ At the Wake Forest University School of Medicine and associated hospital systems, an eFI was integrated into the electronic health record (EHR) in October 2019 (eTables 1 and 2 in Supplement 1). This eFI score is updated on a weekly basis for all patients 55 years or older. The availability of tools such as the eFI is increasingly important in the perioperative realm, as higher frailty scores are indicative of increased postoperative care needs, including hospital length of stay and discharge to transitional care facilities, 30-day readmission rates, and all-cause mortality.^1,3,8^ However, these tools demonstrate substantial heterogeneity in their association with postoperative surgical complication risk; therefore, a closer examination is necessary.^8^

In a previous study, Callahan et al^1^ grouped patients into 3 frailty categories using eFI scores (fit, prefrail, and frail) and found that a patient’s likelihood for worse outcomes increased as the severity of frailty increased.^2,3^ The eFI calculation includes comorbidities, which could also play a role in adverse outcomes (eTables 1 and 2 in Supplement 1). Furthermore, while the eFI has been constructed and validated in an accountable care organization in adults older than 65 years, its association with an exhaustive set of adverse postsurgical and postanesthesia events in a confounder-controlled model has not been tested. To date, only a limited number of patients and a small range of adverse perioperative outcomes have been associated with the eFI at Wake Forest University School of Medicine.

The primary objective of the present investigation was to examine the association of frailty, calculated as eFI at the time of the surgical procedure and categorized as fit (eFI score: ≤0.10), prefrail (eFI score: >0.10 to ≤0.21), or frail (eFI score: >0.21), with adverse events after elective noncardiac surgery. We studied a large diverse cohort of adult patients across a myriad of outcomes specific to the immediate perioperative period in various confounder-controlled models.

## Methods

The Wake Forest University Health Sciences Institutional Review Board deemed this cohort study exempt from ethics review because of its retrospective nature and thus waived the informed consent requirement. We followed the Strengthening the Reporting of Observational Studies in Epidemiology (STROBE) reporting guideline.^9^

### Study Cohort

This single-center, retrospective cohort study was conducted at Atrium Health Wake Forest Baptist Medical Center, a tertiary care, 900-bed university hospital in Winston-Salem, North Carolina. Patients 55 years or older who underwent noncardiac surgical procedures of at least 1 hour in duration between October 1, 2017, and June 30, 2021, were included. We excluded patients with American Society of Anesthesiologists (ASA) Physical Status classification of V (indicating individuals with critical illness not expected to survive without the operation) or VI (indicating individuals with brain death being considered as organ donors) and patients with insufficient historical data to calculate the eFI (eTable 6 in Supplement 1). For patients with multiple noncardiac procedures during the study timeframe, only the first procedure was used.

### eFI Calculation

At Atrium Health Wake Forest Baptist, the eFI is calculated automatically on a weekly basis for patients 55 years or older who had an encounter in the past week, using a look-back window of 2 years. To ensure that an absence of data does not imply robust health status, an eFI score is returned only if there have been at least 2 outpatient encounters with blood pressure measurement in the 2 years prior to the index date in question. For this study, we retrospectively calculated and collected preoperative eFI scores on the day prior to the date of the index operation. In keeping with the timeline of the implementation of a viable EHR and a 2-year look-back period for each eFI calculation, we used the study period starting October 2017 and extended it to June 2021. Although the study is retrospective, the eFI calculation and data extraction, operationalization, and implementation of eFI in the EHR occurred in 2019.

The eFI is derived from 54 deficits, including comorbidities, functional deficits, and laboratory and physical examination findings^2^ (eTables 1 and 2 in Supplement 1) and is reported as a unitless scalar between 0 and 1.0. The eFI calculation is based on the unweighted sum of the score for each deficit divided by the total number of nonmissing items. As in previous work, we classified frailty status as fit, prefrail, and frail.^10^

### Covariates and Confounders

Since adverse surgical outcomes are potentially affected by a wide variety of factors that may confound the association between eFI and these measures, we also extracted 22 potential covariates and confounders for each patient, which included demographics, anesthesia base units (calculated from anesthesia service billing with *Current Procedural Terminology* codes) as a surrogate for surgical intensity, Charlson Comorbidity Index (CCI; score range: 0-24, with 1-2 indicating mild; 3-4 indicating moderate; ≥5 indicating severe comorbidities), and the Area Deprivation Index (ADI) as a measure of socioeconomic disadvantage.^11^ The ADI is a composite measure of 17 US Census variables designed to describe socioeconomic disadvantage based on annual income, educational level, household characteristics, and housing.^11^ The ADI values are presented as national percentile rankings from level 1 to 100, with 1 signifying low level of disadvantage and 100 indicating high level of disadvantage. Patients were stratified into low (ADI <34), medium (ADI 34-65), and high (ADI >65) groups.^12,13^ As the eFI itself included multiple comorbid conditions, we included the CCI to exclude the possibility of eFI being an indicator of adverse outcomes solely based on the presence of coexisting disease. Covariate body mass index data were missing for 374 patients, and ideal body weight data were missing for 538 patients and were addressed using the multiple imputation by chained equation approach.

### Outcomes

The primary outcome was a composite of 1 or more of the following 8 adverse component events: 90-item Patient Safety Indicators (PSI 90) score, hospital-acquired conditions (eTable 9 in Supplement 1), in-hospital mortality, 30-day mortality, 30-day readmission, 30-day emergency department visit after surgery, transfer to a skilled nursing facility after surgery, or unexpected intensive care unit admission after surgery based on planned postoperative disposition at the time of surgical booking (eTables 3 and 4 in Supplement 1).

Secondary outcomes were each of the 8 adverse component events. Furthermore, eFI was used as a continuous variable (in increments of 0.03) and assessed for its association with the same set of both composite and individual outcomes.

### Statistical Analysis

We estimated the association of eFI-based frailty status with the primary outcome and secondary outcomes using logistic regression. We constructed 3 multivariable logistic regression models: model 1 adjusted only for age, sex, and race and ethnicity (minimally adjusted model); model 2 additionally adjusted for CCI to account for comorbidity burden; and model 3 fully adjusted for age, sex, race and ethnicity, CCI, and all other potential confounders and covariates (all of which varied across frailty groups; Table 1).^14^ Race and ethnicity were self-reported (categories: Black; Hispanic; White; and other, including American Indian, American Pacific, Asian, Native American, and others) and were collected in this study to determine any variation in the evolution of frailty status across race and ethnicity. Model 3 included all covariates and confounders in the model, except for 5 variables that did not converge in the logistic regression (length of stay, general anesthesia, blood loss of >100 mL, mean arterial pressure of <65 mm Hg for >5 minutes, and additional operations in the year after the index operation).

**Table 1.** Patient Characteristics Between Electronic Frailty Index (eFI) Groups

Characteristics	eFI group, No. (%)			*P* value
	Fit: eFI ≤0.10 (n = 11 563)	Prefrail: eFI >0.10 to ≤0.21 (n = 15 928)	Frail: eFI >0.21 (n = 5958)	
Age, median (IQR), y	66.0 (60.0-72.0)	68.0 (61.0-74.0)	69.0 (62.0-76.0)	<.001
Sex				
Female	6119 (52.9)	8585 (53.9)	2914 (48.9)	
Male	5444 (47.1)	7343 (46.1)	3044 (51.1)	<.001
Race and ethnicity^a^				
Black	1125 (9.7)	2404 (15.1)	1067 (17.9)	<.001
Hispanic	248 (2.1)	342 (2.1)	99 (1.7)	
White	9902 (85.6)	12 882 (80.9)	4706 (79.0)	
Other^b^	288 (2.5)	300 (1.9)	86 (1.4)	
CCI weighted, median (IQR)	0.00 (0.00-2.00)	2.00 (1.00-3.00)	4.00 (2.00-5.00)	<.001
BMI, median (IQR)	28.30 (24.90-30.84)	29.80 (25.30-32.90)	30.80 (25.70-34.60)	<.001
Insurance				
Governmental	7513 (65.0)	11 993 (75.3)	5179 (86.9)	<.001
Other	4050 (35.0)	3935 (24.7)	779 (13.1)	
Primary language: English	11 326 (98.0)	15 654 (98.3)	5875 (98.6)	.006
North Carolina residence	9943 (86.0)	14 514 (91.1)	5730 (96.2)	<.001
Patient type				
Inpatient	3408 (29.5)	5310 (33.3)	2268 (38.1)	<.001
Outpatient	6068 (52.5)	7840 (49.2)	2894 (48.6)	
Surgery admission	2087 (18.0)	2778 (17.4)	796 (13.4)	
ADI category				
Low level: <34	1223 (10.6)	1236 (7.8)	412 (6.9)	<.001
Medium level: 34-65	5093 (44.0)	7098 (44.6)	2657 (44.6)	
High level: >65	5247 (45.4)	7594 (47.7)	2889 (48.5)	
ADI, median (IQR)	64.00 (50.00-76.00)	66.00 (53.00-76.00)	66.00 (54.00-75.00)	<.001
LOS, median (IQR), d	1.25 (0.28-3.43)	1.47 (0.33-5.05)	2.36 (0.38-7.19)	<.001
ASA PS classification: III or IV^c^	7581 (65.6)	13 746 (86.3)	5802 (97.4)	<.001
General anesthesia	10 023 (86.7)	13 291 (83.4)	4368 (73.3)	<.001
Surgical area				
General	2555 (22.1)	2882 (18.1)	847 (14.2)	<.001
Neurosurgery	1457 (12.6)	1799 (11.3)	533 (8.9)	
Orthopedics	1746 (15.1)	2774 (17.4)	1281 (21.5)	
Urology	1547 (13.4)	2270 (14.3)	610 (10.2)	
Other	4258 (36.8)	6203 (38.9)	2687 (45.1)	
Surgical duration				
<90 min	2139 (18.5)	2899 (18.2)	1203 (20.2)	<.001
90-180 min	4684 (40.5)	6738 (42.3)	2792 (46.9)	
>180 min	4740 (41.0)	6291 (39.5)	1963 (32.9)	
Surgical duration, median (IQR), min	155.00 (102.00-233.00)	152.00 (102.00-231.00)	139.00 (97.00-208.00)	<.001
ABUs, mean (SD)	7.05 (3.66)	7.37 (3.91)	7.15 (3.93)	<.001
Work RVUs, mean (SD)	17.58 (10.85)	17.78 (11.20)	16.40 (11.13)	<.001
EBL: ≥100 mL	2480 (21.4)	3654 (22.9)	1324 (22.2)	.01
≥5 hypotensive min: MAP <65 mm Hg (%)	3026 (26.2)	4267 (26.8)	1543 (25.9)	.40
No. of surgeries within 1 y after discharge, mean (SD)	0.21 (0.60)	0.27 (0.75)	0.34 (0.91)	<.001

Abbreviations: ABUs, anesthesia base units (calculated from anesthesia service billing with *Current Procedural Terminology* codes); ADI, Area Deprivation Index; ASA PS, American Society of Anesthesiologists Physical Status Classification System (classification: I-VI, with VI indicating individuals with brain death being considered as organ donors); BMI, body mass index (calculated as weight in kilograms divided by height in meters squared); CCI, Charlson Comorbidity Index (score range: 0-24, with 1-2 indicating mild; 3-4 indicating moderate; ≥5 indicating severe comorbidities); EBL, estimated blood loss; LOS, length of stay; MAP, mean arterial pressure; RVUs, relative value units.

^a^ Race and ethnicity were self-reported and obtained from the institutional electronic health record.

^b^ Other category included American Indian, American Pacific, Asian, and Native American.

^c^ The burden of illness resides in patients with ASA PS III or IV compared with those with ASA PS I or II.

To avoid multicollinearity, we calculated the variance inflation factor for each covariate in the model, and any collinear covariates were removed if the variance inflation factor was more than 10^15^ (eTable 5 in Supplement 1). Restricted cubic splines were included in each of the continuous variables to relax the linearity assumptions of the models. Splines were removed from variables in which the nonlinear components were not statistically significant. In an exploratory analysis, we treated the eFI as a continuous rather than a categorical variable and performed logistic regression for the composite and individual outcomes. For these analyses, we estimated associations, assuming an increase of 0.03 units in the eFI, which was suggested as a minimally clinically relevant difference in deficit accumulation for frailty indices in general.^16^

We adjusted for multiple comparisons when appropriate using the Holm correction^17^ and otherwise treated *P* < .05 as statistically significant. Statistical analyses were performed in R version 3.6.1 (R Foundation for Statistical Computing) using RStudio environment v1.1.456 (Posit PBC).

## Results

Of the 50 456 patients who underwent noncardiac surgical procedures lasting more than 1 hour at Atrium Health Wake Forest Baptist during the study period, 17 007 were excluded from this analysis, mostly due to the inability to calculate the eFI because outpatient encounters prior to the day of surgery were lacking (eFigure 1 in Supplement 1). Of the 33 449 patients included, there were 17 618 females (52.7%) and 15 831 males (47.3%), with a median (IQR) age of 67 (61-74) years. Patients were classified by their eFI as fit (11 563 [34.6%]), prefrail (15 928 [47.6%]), or frail (5958 [17.8%]). Baseline and demographic characteristics of patients are shown in Table 1. All variables were statistically significant across frailty groups, except for minutes of intraoperative hypotension. Table 1 also shows potential confounders and covariates, including those in the fully adjusted model, and their prevalence by frailty status.

Frailty was associated with increased odds of the composite outcome in all 3 models (Table 2). The C statistic of the models progressively increased with addition of more covariates (0.61, 0.62, and 0.82 for models 1, 2, and 3, respectively), and the Brier statistic progressively decreased (0.224, 0.219, and 0.124, respectively), suggesting that inclusion of more covariates yields more accurate estimates. In comparison with the fit group, the prefrail group had a 9% increase (odds ratio [OR], 1.09; 95% CI, 1.04-1.12) and the frailty group had a 42% increase (OR, 1.42; 95% CI, 1.34-1.53) in odds of the primary composite outcome in the fully adjusted model (Table 2). Using model 2, patients with prefrail (OR, 1.24; 95% CI, 1.18-1.30) and frail (OR, 1.71; 95% CI, 1.58-1.82) statuses were more likely to experience postoperative adverse events compared with patients with a fit status. The ORs and 95% CIs for the composite and individual outcomes are provided in the Figure for model 3 and in eFigure 2 in Supplement 1 for model 2.

**Table 2.** Association of Electronic Frailty Index (eFI) Groups With the Primary Composite Outcome of Adverse Outcomes

Multivariable logistic regression models	eFI Group comparison, OR (95% CI)	
	Prefrail vs fit	Frail vs fit
Model 1 adjusted for age, sex, and race and ethnicity^a^	1.49 (1.43-1.57)	2.75 (2.56-2.94)
Model 2 adjusted for age, sex, race and ethnicity, and CCI	1.24 (1.18-1.30)	1.71 (1.58-1.82)
Model 3 fully adjusted for significant covariates and potential confounders	1.09 (1.04-1.12)	1.42 (1.34-1.53)

Abbreviation: CCI, Charlson Comorbidity Index.

^a^ With splines, nonlinear association.

**Figure.**
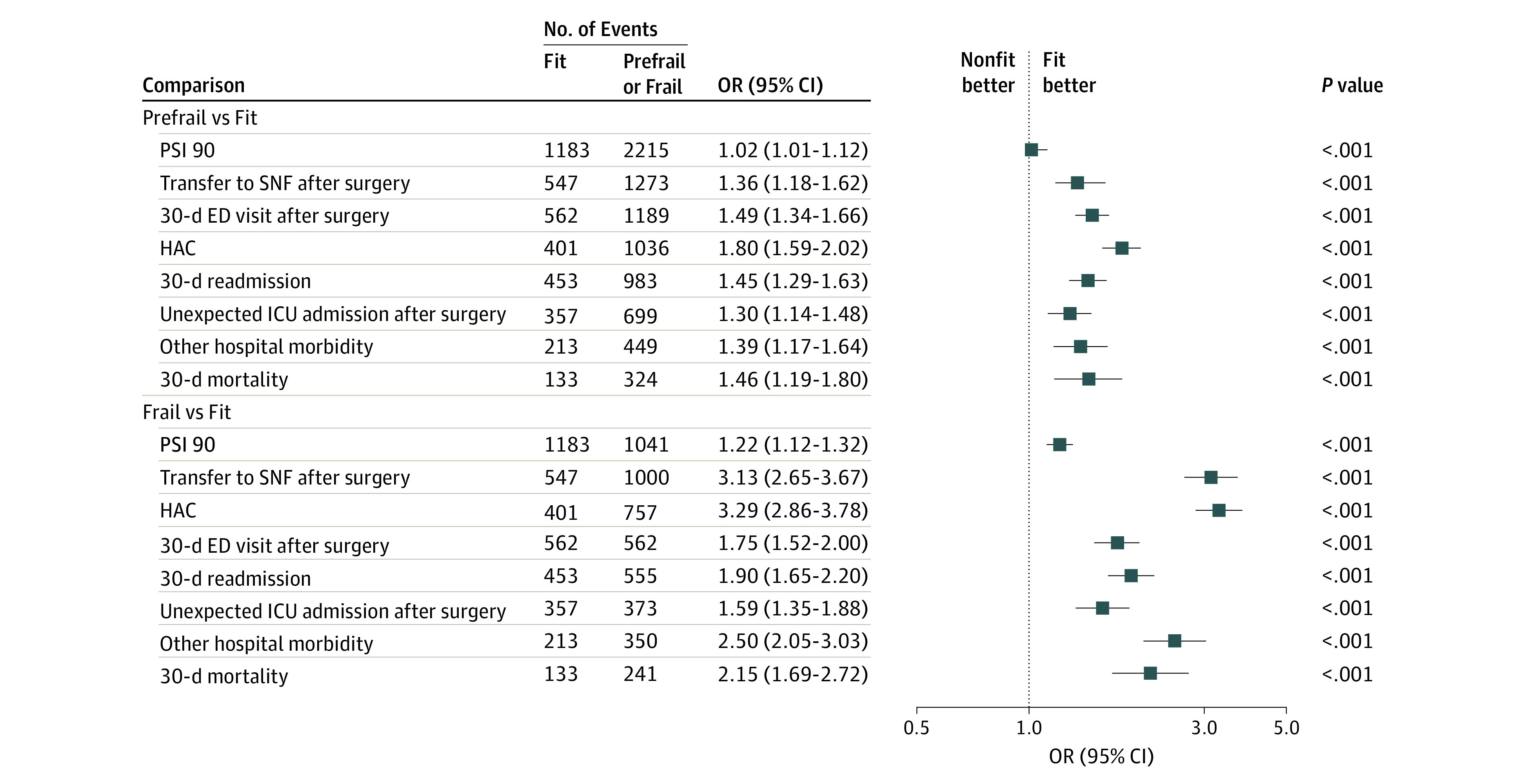
Varying Risk of Study Outcome for Electronic Frailty Index Groups After Adjustment for All Potential Confounders and Covariates ED indicates emergency department; HAC, hospital-acquired condition; ICU, intensive care unit; OR, odds ratio; PSI 90, 90-item Patient Safety Indicators; and SNF, skilled nursing facility.

All individual outcomes were associated with frailty, and most were associated with prefrail status in both models 2 and 3. In analyses treating eFI as a continuous variable, model 3 showed an increase in the odds for the primary composite outcome (OR, 1.06; 95% CI, 1.03-1.13) and all individual components for each increment of 0.03 units in the eFI, except for PSI 90 score (OR, 1.02; 95% CI, 0.91-1.11) (Table 3). Over the range of 0 to 0.3, comprising 97.0% of the patients, the absolute risk of the composite outcome increased linearly with increasing eFI, grouped by increments of 0.03 units (*R*^2^ = 0.66; *P* < .001) (eFigure 3 in Supplement 1) as did a LOWESS (locally weighted scatterplot smoothing) plot of the log OR of the composite outcome and eFI (*R*^2^ = 0.69; *P* < .001) (eFigure 4 in Supplement 1). The absolute event rates for each outcome are shown in eTable 8 in Supplement 1, and the ORs for the unadjusted analyses are shown in eTable 7 in Supplement 1. eTable 10 in Supplement 1 provides the ORs for the covariates of the composite outcome for each variable.

**Table 3.** Adjusted Odds Ratios (ORs) of Outcomes for Frailty in Model 3 With Electronic Frailty Index as a Continuous Variable^a^

Outcome	OR (95% CI)
Primary composite outcome	1.06 (1.03-1.13)
30-d mortality	1.08 (1.04-1.14)
30-d readmission	1.05 (1.02-1.22)
30-d ED visit after surgery	1.09 (1.06-1.17)
Unexpected ICU admission after surgery	1.03 (1.01-1.09)
PSI 90 score	1.02 (0.91-1.11)
HAC	1.33 (1.07-1.65)
Other hospital morbidity	1.19 (1.07-1.28)
Transfer to SNF after surgery	1.22 (1.16-1.37)

Abbreviations: ED, emergency department; HAC, hospital-acquired condition; ICU, intensive care unit; PSI 90, 90-item Patient Safety Indicators; SNF, skilled nursing facility.

^a^ Outcomes are shown for an increase in electronic Frailty Index of 0.03 units.

## Discussion

This retrospective cohort study showed that frailty, as measured by the eFI, was associated with a higher risk of postoperative mortality and major morbidity, even after adjustment for comorbidity burden and multiple other covariates. The findings support the notion that electronically estimated frailty may provide some value in identifying surgical patients at risk for adverse outcomes in the perioperative setting beyond the risks anticipated with age and comorbidity burden alone.

Nearly two-thirds of the cohort was composed of patients at an increased frailty risk, with 17.8% of patients in the frail group and 47.6% in the prefrail group. The observed prevalence of frailty in the present study is comparable to estimates from a 2015 study by Bandeen-Roche and colleagues,^18^ which examined the prevalence of frailty among older adults in the US (15% frail and 45% prefrail). The finding is also similar to that of another analysis using the Hopkins Frailty Score but lower than that using the somewhat outdated Modified Frailty Index (mFI).^19^ Differences in frailty estimates may be due to the broad range and definitions of frailty and study populations among investigations, complicating comparisons across them.^20^

The eFI used in this study is standardized and automatically calculated within the EHR to identify patients at increased risk of adverse outcomes. The value can be displayed in a variety of note types within the EHR. The eFI score itself includes 54 total deficits and is calculated as the unweighted sum of the score for each deficit divided by the total number of nonmissing items. We believe that this score could be implemented in any institution using the same EHR and likely by many other facilities. Furthermore, the components of the eFI include many potentially modifiable risk factors, such as anemia, diabetes, thyroid disease, heart failure, and hypertension, suggesting that preoperative interventions could be beneficial for modifying outcomes of eFI-guided therapy.

There have been a number of frailty instruments used to date, including the mFI,^21^ the modified Hopkins Frailty Index,^22^ and the 14-item Risk Analysis Index (RAI).^7,23^ All of these tools have been associated with adverse patient outcomes in various settings. Despite this growing area of research, each of these tools has limitations. For example, the mFI includes 11 items, 9 of which relate to preexisting comorbidities, limiting its utility to represent the multidimensional nature of frailty. The RAI relies on self-reported surveys completed by the patient or caregiver; these responses are then interpreted by clinicians using their best judgment.^7,24^ However, we are sensitive to the issues raised by a consensus conference at the National Institute on Aging (NIA), suggesting that the various frailty tools should be validated against each other and included in meaningful interventional trials aimed at reducing risk.^20^ The eFI tool we analyzed demonstrates its association with a wide variety of adverse perioperative outcomes, supports the NIA goal, and may be validated against the eFI score in other institutions or in interventional trials aimed at eFI score–based care or outcome improvement.

This study builds on the work of Callahan and colleagues,^1^ who reported the feasibility and utility of the eFI in identifying patients at high risk of adverse outcomes, including a limited sample of perioperative patients.^2^ Consistent with previous studies,^1,3,25,26,27,28,29^ this study found that an increased frailty burden was associated with a higher risk of the primary composite end point (including 30-day mortality) and most of the secondary individual perioperative outcomes. We used previously defined and validated cutoff values of eFI to stratify the patients, but our analysis of the eFI as a continuous variable also demonstrated an increase in the composite outcome.^10,20^ Although various comorbid conditions were elements in the eFI, the results nonetheless suggested that increasing eFI was associated with perioperative risk, even after accounting for comorbidity. We acknowledge, however, that other work has shown a substantial error rate in estimating prolonged hospitalization, readmission rates, and/or postoperative complications using deficit accumulation or phenotype assessments of frailty.^30^ As has been recommended by the NIA consensus conference discussion on frailty measures, such error rates highlight the need to develop better screening tools, with particular focus on modifying relevant outcomes by consideration of the preoperative frailty measure.^20^

The eFI may be used to assess the appropriateness of surgical procedures in patients with prefrail and frail statuses and to identify patients who may benefit from preoperative optimization of modifiable factors contributing to the frailty status. Additionally, findings of this study support the notion that when eFI is treated as a continuous variable and increases in increments of 0.03, there is an incremental increase in the risk of the primary composite end point of 30-day mortality. Given the dynamic nature of the eFI, clinicians may use the eFI score categories, as initially defined by Callahan and colleagues,^1^ at the time of preoperative assessments or use the absolute score over time to determine changes in frailty preceding a surgical episode of care.

### Limitations

This study has some limitations. First, the eFI analyzed in this study represents a snapshot of a patient’s frailty and is calculated on the basis of a patient’s presentation prior to the surgical procedure during preoperative assessment. For the purposes of this investigation, we used the date of surgery itself as the date of such assessment, understanding that decision-making regarding the operation or preoperative optimization interventions was made well in advance of the encounter. Second, frailty is a dynamic state, and the study design prevented us from drawing conclusions regarding the time-dependent nature of a given patient’s eFI score. Third, the study was confined to a single institution, although it involved a diverse surgical population. Future work should validate the findings in other institutions and examine the trajectory of change in the eFI as a factor in perioperative outcomes. Ultimately, the true value of the eFI will be realized only by studying the interventions to address modifiable components of the score or modification in surgical planning and their association with outcome. Fourth, we provided no details of the operationalization of eFI at Atrium Health Wake Forest Baptist or a blueprint for such deployment at other institutions.

## Conclusions

Among patients who underwent noncardiac surgery at a single tertiary care center, frailty (as measured by an automated score derived from EHR data) was associated with an increased risk of a primary composite end point of 30-day mortality and several other adverse perioperative outcomes in the acute care and several other perioperative outcomes in the acute care (30-day readmission, 30-day emergency room visit, in-hospital mortality, 90-item Patient Safety Indicator, hospital-acquired condition, unexpected intensive care unit admission postoperatively) and the delayed (transfer to a skilled nursing facility) postoperative period after controlling for multiple confounders and covariates. Deployment of eFI tools may help with easy screening and possible risk modification, especially in patients who will undergo high-risk surgery.

## Notes

Supplement 1. **eTable 1.** The Original Construction of the Electronic Frailty Index (eFI) Spanned 54 Deficits **eTable 2.** Modifications to the Electronic Frailty Index (eFI) Since Its Original Publication in Pajewski et al **eTable 3.** Definition of Primary Composite Outcome **eTable 4.** Center for Medicare and Medicaid Services (CMS) Patient Safety Indicator 90 (PSI 90), Hospital-Acquired Conditions (HAC) and Other Hospital Morbidity Definitions **eTable 5.** Variance Inflation Factor (VIF) for Estimation of Collinearity Between Covariates **eTable 6.** Analysis of Excluded Patients Where eFI Was Not Calculated Since They Did Not Meet the Criteria for 2 Blood Pressure Readings in the Last Two Years **eTable 7.** Odds of Outcomes Across Frail Groups, Adjusted for Age, Sex and Race **eTable 8.** Event Rates for Study Outcomes by Electronic Frailty Index (eFI) Groups **eTable 9.** Hospital Acquired Conditions (HAC) per CMS Definition and Distribution in the Study Cohort **eTable 10.** Odds Ratios of Covariates for the Composite Outcome in a Multivariable Logistic Regression Adjusted for Significant Covariates and Charlson Comorbidity Index **eFigure 1.** Flow Diagram for the Study Population **eFigure 2.** Box Plot Showing Varying Risk of Outcomes for Electronic Frailty Index (eFI) Groups After Adjustment for Age, Gender, Race, and Charlson Comorbidity Index (CCI) **eFigure 3.** Absolute Risk of the Composite Outcome With Increasing eFI, Grouped by Increments of 0.03 **eFigure 4.** LOWESS Plot for Log Odds of Composite Outcome and eFI as a Continuous Variable

Supplement 2.
Data Sharing Statement

## References

[1] Callahan KE, Clark CJ, Edwards AF, . Automated frailty screening at-scale for pre-operative risk stratification using the electronic frailty index. J Am Geriatr Soc. 2021;69(5):1357-1362. doi:10.1111/jgs.17027 33469933 PMC8127394

[2] Pajewski NM, Lenoir K, Wells BJ, Williamson JD, Callahan KE. Frailty screening using the electronic health record within a Medicare accountable care organization. J Gerontol A Biol Sci Med Sci. 2019;74(11):1771-1777. doi:10.1093/gerona/glz017 30668637 PMC6777083

[3] Stutsrim AE, Brastauskas IM, Craven TE, . Automated electronic frailty index is associated with non-home discharge in patients undergoing open revascularization for peripheral vascular disease. Am Surg. Published online August 16, 2022;31348221121547. doi:10.1177/00031348221121547 35971786 PMC11459651

[4] Walston J, Buta B, Xue QL. Frailty screening and interventions: considerations for clinical practice. Clin Geriatr Med. 2018;34(1):25-38. doi:10.1016/j.cger.2017.09.004 29129215 PMC5726589

[5] Dent E, Kowal P, Hoogendijk EO. Frailty measurement in research and clinical practice: a review. Eur J Intern Med. 2016;31:3-10. doi:10.1016/j.ejim.2016.03.007 27039014

[6] Levit LA, Kaltenbaugh MW, Magnuson A, . Challenges and opportunities to developing a frailty index using electronic health record data. J Geriatr Oncol. 2021;12(5):851-854. doi:10.1016/j.jgo.2021.02.008 33622653 PMC12478465

[7] Hall DE, Arya S, Schmid KK, . Development and initial validation of the risk analysis index for measuring frailty in surgical populations. JAMA Surg. 2017;152(2):175-182. doi:10.1001/jamasurg.2016.4202 27893030 PMC7140150

[8] Le ST, Liu VX, Kipnis P, Zhang J, Peng PD, Cespedes Feliciano EM. Comparison of electronic frailty metrics for prediction of adverse outcomes of abdominal surgery. JAMA Surg. 2022;157(5):e220172. doi:10.1001/jamasurg.2022.0172 35293969 PMC8928095

[9] von Elm E, Altman DG, Egger M, Pocock SJ, Gøtzsche PC, Vandenbroucke JP; STROBE Initiative. Strengthening the Reporting of Observational Studies in Epidemiology (STROBE) statement: guidelines for reporting observational studies. BMJ. 2007;335(7624):806-808. doi:10.1136/bmj.39335.541782.AD 17947786 PMC2034723

[10] Hoover M, Rotermann M, Sanmartin C, Bernier J. Validation of an index to estimate the prevalence of frailty among community-dwelling seniors. Health Rep. 2013;24(9):10-17. 24258362

[11] Maroko AR, Doan TM, Arno PS, Hubel M, Yi S, Viola D. Integrating social determinants of health with treatment and prevention: a new tool to assess local area deprivation. Prev Chronic Dis. 2016;13:E128. doi:10.5888/pcd13.160221 27634778 PMC5027849

[12] Kind AJH, Buckingham WR. Making neighborhood-disadvantage metrics accessible - the Neighborhood Atlas. N Engl J Med. 2018;378(26):2456-2458. doi:10.1056/NEJMp1802313 29949490 PMC6051533

[13] The Neighborhood Atlas. Area deprivation index. University of Wisconsin School of Medicine and Public Health. Accessed April 28, 2022. https://www.neighborhoodatlas.medicine.wisc.edu/

[14] Dunkler D, Plischke M, Leffondré K, Heinze G. Augmented backward elimination: a pragmatic and purposeful way to develop statistical models. PLoS One. 2014;9(11):e113677. doi:10.1371/journal.pone.0113677 25415265 PMC4240713

[15] Vittinghoff E, Glidden DV, Shiboski SC, McCulloch CE. Regression Methods in Biostatistics: Linear, Logistic, Survival, and Repeated Measures Models. 2nd ed. Springer; 2012. doi:10.1007/978-1-4614-1353-0

[16] Theou O, van der Valk AM, Godin J, . Exploring clinically meaningful changes for the frailty index in a longitudinal cohort of hospitalized older patients. J Gerontol A Biol Sci Med Sci. 2020;75(10):1928-1934. doi:10.1093/gerona/glaa084 32274501 PMC7518565

[17] Bonferroni CE. Teoria Statistica delle Classi e Calcolo delle Probabilità. Seeber; 1936.

[18] Bandeen-Roche K, Seplaki CL, Huang J, . Frailty in older adults: a nationally representative profile in the United States. J Gerontol A Biol Sci Med Sci. 2015;70(11):1427-1434. doi:10.1093/gerona/glv133 26297656 PMC4723664

[19] Weller RS, Foard KL, Harwood TN. Evaluation of a wireless, portable, wearable multi-parameter vital signs monitor in hospitalized neurological and neurosurgical patients. J Clin Monit Comput. 2018;32(5):945-951. doi:10.1007/s10877-017-0085-0 29214598

[20] Walston J, Bandeen-Roche K, Buta B, . Moving frailty toward clinical practice: NIA intramural frailty science symposium summary. J Am Geriatr Soc. 2019;67(8):1559-1564. doi:10.1111/jgs.15928 31045254 PMC6830521

[21] Arya S, Long CA, Brahmbhatt R, . Preoperative frailty increases risk of nonhome discharge after elective vascular surgery in home-dwelling patients. Ann Vasc Surg. 2016;35:19-29. doi:10.1016/j.avsg.2016.01.052 27263810

[22] Mrdutt MM, Papaconstantinou HT, Robinson BD, Bird ET, Isbell CL. Preoperative frailty and surgical outcomes across diverse surgical subspecialties in a large health care system. J Am Coll Surg. 2019;228(4):482-490. doi:10.1016/j.jamcollsurg.2018.12.036 30885474

[23] Varley PR, Borrebach JD, Arya S, . Clinical utility of the risk analysis index as a prospective frailty screening tool within a multi-practice, multi-hospital integrated healthcare system. Ann Surg. 2021;274(6):e1230-e1237. doi:10.1097/SLA.0000000000003808 32118596

[24] Gilbert T, Neuburger J, Kraindler J, . Development and validation of a hospital frailty risk score focusing on older people in acute care settings using electronic hospital records: an observational study. Lancet. 2018;391(10132):1775-1782. doi:10.1016/S0140-6736(18)30668-8 29706364 PMC5946808

[25] Beggs T, Sepehri A, Szwajcer A, Tangri N, Arora RC. Frailty and perioperative outcomes: a narrative review. Can J Anaesth. 2015;62(2):143-157. doi:10.1007/s12630-014-0273-z 25420470

[26] Birkelbach O, Mörgeli R, Spies C, . Routine frailty assessment predicts postoperative complications in elderly patients across surgical disciplines - a retrospective observational study. BMC Anesthesiol. 2019;19(1):204. doi:10.1186/s12871-019-0880-x 31699033 PMC6839249

[27] Shinall MC Jr, Arya S, Youk A, . Association of preoperative patient frailty and operative stress with postoperative mortality. JAMA Surg. 2020;155(1):e194620. doi:10.1001/jamasurg.2019.4620 31721994 PMC6865246

[28] Siddiqui E, Banco D, Berger JS, Smilowitz NR. Frailty assessment and perioperative major adverse cardiovascular events after noncardiac surgery. Am J Med. 2023;136(4):372-379.e5. doi:10.1016/j.amjmed.2022.12.033 36657557 PMC10038881

[29] Hewitt J, Carter B, McCarthy K, . Frailty predicts mortality in all emergency surgical admissions regardless of age. An observational study. Age Ageing. 2019;48(3):388-394. doi:10.1093/ageing/afy217 30778528

[30] Sonny A, Kurz A, Skolaris LA, . Deficit accumulation and phenotype assessments of frailty both poorly predict duration of hospitalization and serious complications after noncardiac surgery. Anesthesiology. 2020;132(1):82-94. doi:10.1097/ALN.0000000000002959 31834870

