# Supplementary material for: Automated Electronic Frailty Index–Identified Frailty Status and Associated Postsurgical Adverse Events
Source: JAMA Netw Open. 2023 Nov 6;6(11):e2341915. doi: 10.1001/jamanetworkopen.2023.41915 (PMC10628731; doi:10.1001/jamanetworkopen.2023.41915)

Supplemental Online Content

Khanna AK, Motamedi V, Bouldin B, et al. Automated electronic frailty index–identified frailty status and associated postsurgical adverse events. *JAMA Netw Open*. 2023;6(11):e2341915. doi:10.1001/jamanetworkopen.2023.41915

eTable 1. The Original Construction of the Electronic Frailty Index (eFI) Spanned 54 Deficits

eTable 2. Modifications to the Electronic Frailty Index (eFI) Since Its' Original Publication in Pajewski et al

eTable 3. Definition of Primary Composite Outcome

eTable 4. Center for Medicare and Medicaid Services (CMS) Patient Safety Indicator 90 (PSI 90), Hospital-Acquired Conditions (HAC) and Other Hospital Morbidity Definitions

eTable 5. Variance Inflation Factor (VIF) for Estimation of Collinearity Between Covariates

eTable 6. Analysis of Excluded Patients Where eFI Was Not Calculated Since They Did Not Meet the Criteria for 2 Blood Pressure Readings in the Last Two Years

eTable 7. Odds of Outcomes Across Frail Groups, Adjusted for Age, Sex and Race

eTable 8. Event Rates for Study Outcomes by Electronic Frailty Index (eFI) Groups

eTable 9. Hospital Acquired Conditions (HAC) per CMS Definition and Distribution in the Study Cohort

eTable 10. Odds Ratios of Covariates for the Composite Outcome in a Multivariable Logistic Regression Adjusted for Significant Covariates and Charlson Comorbidity Index

eFigure 1. Flow Diagram for the Study Population

eFigure 2. Box Plot Showing Varying Risk of Outcomes for Electronic Frailty Index (eFI) Groups After Adjustment for Age, Gender, Race, and Charlson Comorbidity Index (CCI)

© 2023 Khanna AK et al. *JAMA Network Open*

eFigure 3. Absolute Risk of the Composite Outcome With Increasing eFI, Grouped by Increments of 0.03

eFigure 4. LOWESS Plot for Log Odds of Composite Outcome and eFI as a Continuous Variable

This supplemental material has been provided by the authors to give readers additional information about their work.

eTable 1. The original construction of the electronic frailty index (eFI) spanned 54 deficits.

The general areas covered by these deficits are shown in the first table below. Details, including cutoffs for laboratory measures and diagnosis code lists are described in Pajewski *et al.*

Area	Characteristic	Area	Characteristic
Morbidity	Anemia	Vital Signs and Laboratory Measures	Body Mass Index
	Atrial Fibrillation		Blood Pressure
	Stroke/TIA		Kidney Function
	Kidney Disease		Glucose
	Diabetes		Cholesterol
	Fragility or Fracture		Triglycerides
	Heart Failure		Potassium
	Valvular Disease		Sodium
	Hypertension		Liver Function
	Hypotension/Syncope		Mean Corpuscular Volume
	Heart Disease		Blood Urea Nitrogen
	Osteoporosis		Calcium
	Pulmonary Disease		Albumin
	Thyroid Disease		Total Protein
	Urinary Incontinence / Disease	Cognition and Mood	Dementia / Cognitive Problems
	Liver Disease		Delirium
	Cancer		Depression
	Peptic Ulcer		Stress
	Skin Ulcer		Self-Reported General Health
Function	Arthritis	Sensory Loss	Hearing Impairment
	Falls		Visual Impairment
	Activity Limitation	Other	Weight Loss
	Peripheral Vascular Disease		Chronic Pain
	Parkinson's disease		Polypharmacy
	Dizziness or Vertigo		Smoking
	Dyspnea		

© 2023 Khanna AK et al. *JAMA Network Open*

eTable 2. Modifications to the electronic frailty index (eFI) since its' original publication in Pajewski et al.

Change	Diagnosis Code Listing (if applicable)	References
Addition of deficit for diagnosed delirium	A81.2, E51.2, F05, F10.121, F10.221, F10.231, F10.921, F11.121, F11.221, F11.921, F12.121, F12.221, F12.921, F13.121, F13.221, F12.231, F13.921, F13.931, F14.121, F14.221, F14.921, F15.121, F15.221, F15.921, F16.121, F16.221, F16.921, F18.121, F18.221, F18.921, F19.121, F19.221, F19.231, F19.921, F19.931, G04.3x, G92, G93.4x, I67.3, I67.4, I67.83, J10.81, J11.81, P91.6x,	Kim DH, Lee J, Kim CA, Huybrechts KF, Bateman BT, Patorno E, Marcantonio ER. Evaluation of algorithms to identify delirium in administrative claims and drug utilization database. Pharmacoepidemiol Drug Saf. 2017 Aug;26(8):945-953.
Modification of diagnosis codes for dementia	A81.0x, F01.5x, F02.8x, F03.9x, F06.8, F10.27, F19.97, G10, G11.9, G23.x, G30.x, G31,0, G31.1, G31.2, G31.83, G31.89, G90.3, I67.3, R41.3	
Changed calculation of estimated glomerular filtration rate to the race-free estimating equations		Inker LA, Eneanya ND, Coresh J, et al. New Creatinine- and Cystatin C-Based Equations to Estimate GFR without Race. N Engl J Med. 2021;385(19):1737-1749.

© 2023 Khanna AK et al. *JAMA Network Open*

Broadening of diagnosis code listing for visual impairment deficit	H3530, H35.31x, H35.32x, H35.35x, H40.02x, H40.06x, H40.10Xx, H40.11x, H40.12x, H40.13x, H40.14x, H40.20Xx, H40.21x, H40.22x, H40.23x, H40.24x, H40.30Xx, H40.31Xx, H40.32Xx, H40.33Xx, H40.40Xx, H40.41Xx, H40.42Xx, H40.43Xx, H40.50Xx, H40.51Xx, H40.52Xx, H40.53Xx, H40.60Xx, H40.61Xx, H40.62Xx, H40.63Xx, H40.81x, H40.82x, H40.89, H40.9, H42, H47.61x, H53.04x, H53.30, H53.34, H53.4x, H53.60, H53.61, H53.62, H53.69, H53.7x, H53.8, H53.9, H54.0x, H54.10, H54.11x, H54.12x, H54.2x, H54.3, H54.4x, H54.5x, H54.6x, H54.7, H54.8	Cheng D, DuMontier C, Yildirim C, et al. Updating and Validating the U.S. Veterans Affairs Frailty Index: Transitioning From ICD-9 to ICD-10. *J Gerontol A Biol Sci Med S*ci. 2021;76(7):1318-1325.

eTable 3. Definition of primary composite outcome

Primary outcome is a composite of:
1. 30-day mortality
2. 30-day readmission
3. 30-day ED visit after surgery
4. Unexpected ICU admission after surgery
5. PSI 90
6. HAC CMS
7. Other hospital morbidity
8. Transfer to SNF after surgery

CMS: Centers for Medicare & Medicaid Services
ED: Emergency department
HAC: Hospital-acquired condition
ICU: Intensive care unit
PSI 90: Center for Medicare and Medicaid Services Patient Safety Indicator 90
SNF: Skilled nursing facility

eTable 4: Center for Medicare and Medicaid Services (CMS) Patient Safety Indicator 90 (PSI 90), Hospital-Acquired Conditions (HAC) and other hospital morbidity definitions

**Safety Indicator**	**Quality Indicator**	**References**
**PSI 90**		
Pressure ulcer	PSI03 Pressure Ulcer	https://qualityindicators.ahrq.gov/measures/PSI_TechSpec
Latrogenic pneumothorax	PSI 06 Iatrogenic Pneumothorax	
Central venous catheter-related blood stream infection	PSI 07 Central Venous Catheter-Related Blood Stream	
In-hospital fall with hip fracture	PSI 08 In Hospital Fall with Hip Fracture	
Perioperative pulmonary embolism	PSI 12 Perioperative Pulmonary Embolism or Deep Vein Thrombosis	
Postoperative sepsis	PSI 13 Postoperative Sepsis	
Postoperative wound dehiscence	PSI 14 Postoperative Wound Dehiscence	
Abdominal pelvic accidental puncture or laceration	PSI 15 Abdominopelvic Accidental Puncture or Laceration	
**Hospital Acquired Condition (CMS Definition)**		
Foreign object retained after surgery	HAC 01	https://www.cms.gov/Medicare/Medicare-Fee-for-Service-Payment/HospitalAcqCond/Hospital-Acquired_Conditions
Air embolism	HAC 02	
Blood incompatibility	HAC 03	
Stage III and IV pressure ulcers	HAC 04	
Falls and trauma	HAC 05	
Catheter-associated urinary tract infection	HAC 06	
Vascular catheter-associated infection	HAC 07	
Surgical site infection-mediastinitis after coronary bypass graft (CABG)	HAC 08	
Manifestations of poor glycemic control	HAC 09	
Deep vein thrombosis (DVT) / pulmonary embolism (Pe0 with total knee or hip replacement	HAC 10	
Surgical site infection – bariatric surgery	HAC 11	
Surgical site infection – certain orthopedic procedures of spine, shoulder, and elbow	HAC 12	
Surgical site infection (SSI) following cardiac implantable device (CIED) procedures	HAC 13	
Iatrogenic pneumothorax w/ venous catheterization	HAC 14	
**Additional In-hospital Morbidity**		
**Type**	**Name**	**ICD 10 Code**
Urinary	Urinary Complications	N32.9 , N39.9
Urinary	Acute Kidney Injury	N17. 9
Bleeding	Hemorrhage of Hematona or Seroma Complications	L76. 0, L76. 3

© 2023 Khanna AK et al. *JAMA Network Open*

Postoperative Infection	Postoperative Infections	T81. 4
Respiratory	TRALI	J95. 84
Respiratory	Respiratory Complications	J80–J84, J85–J86, J90–J94, J95, J96–J99
Respiratory	Ventilator Associated pneumonia	J95. 851
Respiratory	Other respiratory complications	J00–J06, J10–J18 , J20–J22 , J30–J39 , J40–J47, J60–J70
Cardiovascular Disorders	Acute Myocardial Infarction	I21

© 2023 Khanna AK et al. *JAMA Network Open*

eTable 5: Variance inflation factor (VIF) for estimation of collinearity between covariates.

Covariates	VIF
**Age**	8.9
**Male**	2.7
**Race (ref: White)**	
Black	1.4
Hispanic	1.6
Other	1.2
**IBW**	2.6
**Body Mass Index**	5.9
**Insurance (ref: Governmental)**	
Other	1.4
**Primary Language, English**	1.6
**Instate**	1.1
**Patient type (ref: Outpatient)**	
Inpatient	1.5
Surgery Admit	1.2
**Area Deprivation Index**	4.5
**CCI Weighted**	4.4
**Length Of Stay**	-
**ASA PS 3 or 4**	1.1
**General Anesthesia**	-
**Surgical Area (ref: Other)**	
General	1.8
Neurosurgery	1.6
Orthopedics	2.2
Urology	1.6
**Surgical Duration in minutes**	6.4
**Anesthesia Base Units**	4.3
**Work RVU**	5
**Estimated Blood Loss, 100ml or more**	-
**5 or more hypotensive minutes, mean arterial pressure <65 mmHg**	-

© 2023 Khanna AK et al. *JAMA Network Open*

Number of Surgeries within 1 Year after discharge	-
**eFi Group (ref: Fit)**	
Pre-Frail	1.7
Frail	2.1

© 2023 Khanna AK et al. *JAMA Network Open*

eTable 6: Analysis of excluded patients where eFI was not calculated since they did not meet the criteria for 2 blood pressure readings in the last two years.

13304 excluded for missing eFI because of lack of repeated blood pressures.

	Odds Ratio	
	Pre-Frail vs Fit	Frail vs Fit
Composite	1.04 (95% CI : 0.86 - 1.12)	1.46 (95% CI : 1.21 - 1.62)

Overall, 46753 patients (included and excluded)

	Odds Ratio	
	Pre-Frail vs Fit	Frail vs Fit
Composite	1.08 (95% CI : 1.02 -1.26)	1.68 (95% CI : 1.44 - 1.95)

© 2023 Khanna AK et al. *JAMA Network Open*

eTable 7: Odds of outcomes across frail groups, adjusted for age, sex and race.

	Pre-Frail vs Fit	Frail vs Fit
Primary Composite Outcome	1.51 (95% CI: 1.44 - 1.59)	2.76 (95% CI: 2.58 – 2.95)
Transfer to SNF after Surgery	1.58 (95% CI: 1.43 - 1.76)	3.34 (95% CI: 2.98 – 3.74)
Unexpected ICU admission after Surgery	1.42 (95% CI: 1.25 - 1.62)	2.07 (95% CI: 1.78 - 2.40)
30 Day Mortality	1.67 (95% CI: 1.36 - 2.05)	3.28 (95% CI: 2.64 - 4.07)
30 Day Readmission	1.60 (95% CI: 1.42 - 1.79)	2.51 (95% CI: 2.20 - 2.86)
30 Day ED Visit after surgery	1.56 (95% CI: 1.41 - 1.73)	2.02 (95% CI: 1.78 - 2.28)
PSI 90	1.37 (95% CI: 1.29 - 1.45)	2.17 (95% CI: 2.01 - 2.33)
HAC CMS	1.90 (95% CI: 1.69 - 2.14)	3.94 (95% CI: 3.48 - 4.48)
Other hospital morbidity	1.54 (95% CI: 1.30 - 1.82)	3.39 (95% CI: 2.84 – 4.04)

CMS: Centers for Medicare & Medicaid Services
ED: Emergency department
HAC: Hospital-acquired condition
ICU: Intensive care unit
PSI 90: Center for Medicare and Medicaid Services Patient Safety Indicator 90
SNF: Skilled nursing facility

© 2023 Khanna AK et al. *JAMA Network Open*

eTable 8: Event rates for study outcomes by electronic frailty index (eFI) groups

	**Fit (eFI ≤ 0.10) (N= 11,563)**	**Pre-Frail (0.10 < eFI ≤ 0.21) (N= 15,928)**	**Frail (eFI>0.21) (N= 5,958)**	**Overall (N=33,449)**
Primary composite outcome	1250 (10.8)	2419 (15.4)	1151 (19.3)	4820(14.4)
Transfer to SNF after surgery	547 (4.7)	1273 (8.0)	1000 (16.8)	28020 (8.4)
Unexpected ICU admission after surgery	357 (3.1)	699 (4.4)	373 (6.3)	1429(4.3)
30-day mortality	133 (1.2)	324 (2.0)	241 (4.0)	698(2.1)
30-day readmission	453 (3.9)	983 (6.2)	555 (9.3)	1991(5.9)
30-day ED visit after surgery	562 (4.9)	1189 (7.5)	562 (9.4)	2313(6.9)
PSI 90	1183 (10.3)	2215 (14.1)	1041 (17.4)	4439(13.3)
HAC CMS	401 (3.5)	1036 (6.5)	757 (12.7)	2194(6.6)
Other hospital morbidity	213 (1.8)	449 (2.8)	350 (5.9)	1012 (3.0)

CMS: Centers for Medicare & Medicaid Services
ED: Emergency department
HAC: Hospital-acquired condition
ICU: Intensive care unit
PSI 90: Center for Medicare and Medicaid Services Patient Safety Indicator 90
SNF: Skilled nursing facility

© 2023 Khanna AK et al. *JAMA Network Open*

eTable 9: Hospital acquired conditions (HAC) per CMS definition and distribution in the study cohort

HAC	Description	Fit	Pre-frail	Frail	Grand Total
HAC 01	FOREIGN OBJECT RETAINED AFTER SURGERY	1	3	1	5
HAC 02	AIR EMBOLISM	0	1	0	1
HAC 03	BLOOD INCOMPATIBILITY	42	53	45	140
HAC 04	STAGE III AND IV PRESSURE ULCERS	85	451	258	794
HAC 05	FALLS AND TRAUMA	62	84	78	224
HAC 06	CATHETER-ASSOCIATED URINARY TRACT INFECTION (UTI)	117	304	236	657
HAC 07	VASCULAR CATHETER-ASSOCIATED INFECTION	63	67	74	204
HAC 08	SURGICAL SITE INFECTION-MEDIASTINITIS AFTER CORONARY BYPASS GRAFT (CABG)	2	1	0	3
HAC 09	MANIFESTATIONS OF POOR GLYCEMIC CONTROL	1	24	11	36
HAC 11	SURGICAL SITE INFECTION - BARIATRIC SURGERY	28	48	54	130
**Grand Total**		**401**	**1036**	**757**	**2194**

© 2023 Khanna AK et al. *JAMA Network Open*

eTable 10: Odds ratios of covariates for the Composite Outcome in a Multivariable logistic regression adjusted for significant covariates and Charlson Comorbidity Index (CCI)

	Odds Ratio	p Value
**Age#**	0.89 (95% CI : 0.83 - 0.94)	<0.001
**Male**	0.97 (95% CI : 0.87-1.07)	0.53
**Race (ref: White)**		
Black	1.05 (95% CI : 1.01-1.16)	0.003
Hispanic	0.94 (95% CI : 0.71-1.24)	0.16
Other	1.28 (95% CI : 1.02-1.6)	0.37
**IBW**	0.98 (95% CI : 0.9-1.07)	0.46
**Body Mass Index #**	0.92 (95% CI : 0.86-1.03)	0.06
**Insurance (ref: Governmental)**		
Other	0.87 (95% CI : 0.71-1.04)	0.58
**Primary Language, English**	1.27 (95% CI : 0.95-1.69)	0.11
**Instate**	0.97 (95% CI : 0.86-1.09)	0.54
**Patient type (ref: Outpatient)**		
Inpatient	1.06 (95% CI : 1.01-1.19)	0.001
Surgery Admit	1.05 (95% CI : 0.94-1.16)	0.22
**Area Deprivation Index #**	1.01 (95% CI : 0.95-1.05)	0.05
**CCI Weighted**	1.02 (95% CI : 0.94-1.11)	0.07
**Length Of Stay**	-	
**ASA PS 3 or 4**	0.98 (95% CI : 1.03-0.92)	0.08
**General Anesthesia ***	-	-
**Surgical Area (ref: Other)**		
General	1.05 (95% CI : 0.93-1.17)	0.06
Neurosurgery	1.05 (95% CI : 1.02-1.07)	<0.001
Orthopedics	1.07 (95% CI : 1.03-1.11)	<0.001
Urology	1.04 (95% CI : 0.96-1.13)	0.35
**Surgical Duration in minutes**	1.04 (95% CI : 1.02-1.09)	0.001
**Anesthesia Base Units #**	0.96 (95% CI : 0.9-1.03)	0.03
**Work RVU#**	0.83 (95% CI : 0.77-1.02)	0.47
**Estimated Blood Loss, 100ml or more ***	-	
**5 or more hypotensive minutes, mean arterial pressure <65 mmHg ***	-	-
**Number of Surgeries within 1 Year after discharge ***	-	
**eFi Group (ref: Fit)**		
Pre-Frail	1.09 (95% CI : 1.04-1.12)	<0.001

© 2023 Khanna AK et al. *JAMA Network Open*

Frail	1.42 (95% CI : 1.34-1.53)	<0.001

with splines, non-linear association * did not converge

© 2023 Khanna AK et al. *JAMA Network Open*

eFigure 1: Flow diagram for the study population.

ASA: American Society of Anesthesiologists; eFI: electronic frailty index

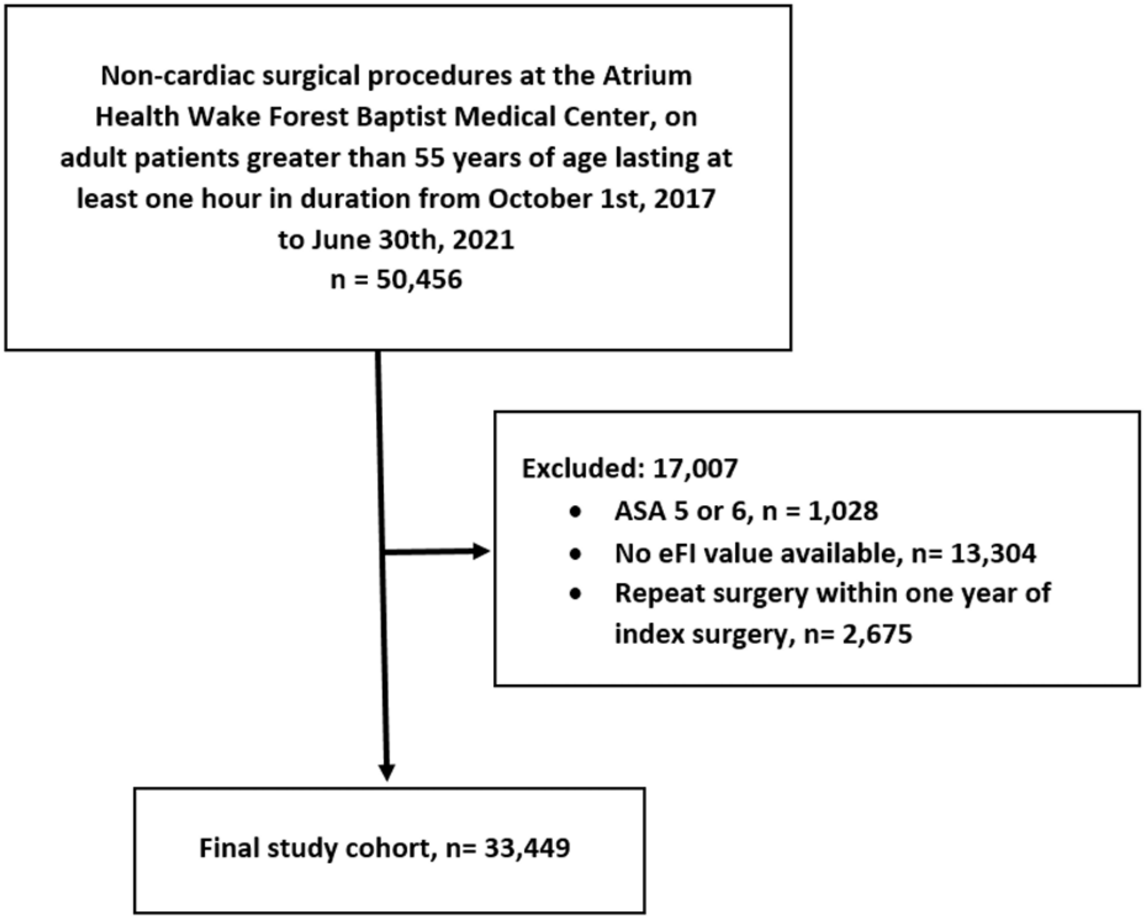

© 2023 Khanna AK et al. *JAMA Network Open*

eFigure 2: Box plot showing varying risk of outcomes for electronic frailty index (eFI) groups after adjustment for age, gender, race, and Charlson Comorbidity Index (CCI).

CCI: Charlson Comorbidity Index; CI: confidence interval; CMS: Centers for Medicare & Medicaid Services; ED: emergency department; HAC: hospital-acquired condition; ICU: intensive care unit; PSI 90: Center for Medicare and Medicaid Services Patient Safety Indicator 90; SNF: skilled nursing facility. Asterisks indicate statistical significance at P<0.05, after Holm correction for multiple comparisons.

Comparison	Outcome	Number of events	Number of events	OR (95% CI)	p–value
**Pre–frail vs Fit**		**Fit**	**Pre–frail**		
	PSI 90	1183	2215	1.36(1.28–1.45)	<0.001
	Transfer to SNF after Surgery	547	1273	1.58(1.43–1.76)	<0.001
	30 Day ED Visit after surgery	562	1189	1.56(1.4–1.73)	<0.001
	HAC CMS	401	1036	1.94(1.69–2.14)	<0.001
	30 Day Readmission	453	983	1.59(1.42–1.79)	<0.001
	Unexpected ICU admission after Surgery	357	699	1.43(1.25–1.62)	<0.001
	Other Hospital Morbidity	213	449	1.54(1.31–1.82)	<0.001
	30 Day Mortality	133	324	1.67(1.36–2.04)	<0.001
**Frail vs Fit**		**Fit**	**Frail**		
	PSI 90	1183	1041	2.16(2.01–2.33)	<0.001
	Transfer to SNF after Surgery	547	1000	3.34(2.99–3.74)	<0.001
	HAC CMS	401	757	3.94(3.47–4.47)	<0.001
	30 Day ED Visit after surgery	562	562	2.01(1.78–2.28)	<0.001
	30 Day Readmission	453	555	2.51(2.02–2.85)	<0.001
	Unexpected ICU admission after Surgery	357	373	2.07(1.78–2.41)	<0.001
	Other Hospital Morbidity	213	350	3.38(2.84–4.03)	<0.001
	30 Day Mortality	133	241	3.27(2.63–4.07)	<0.001

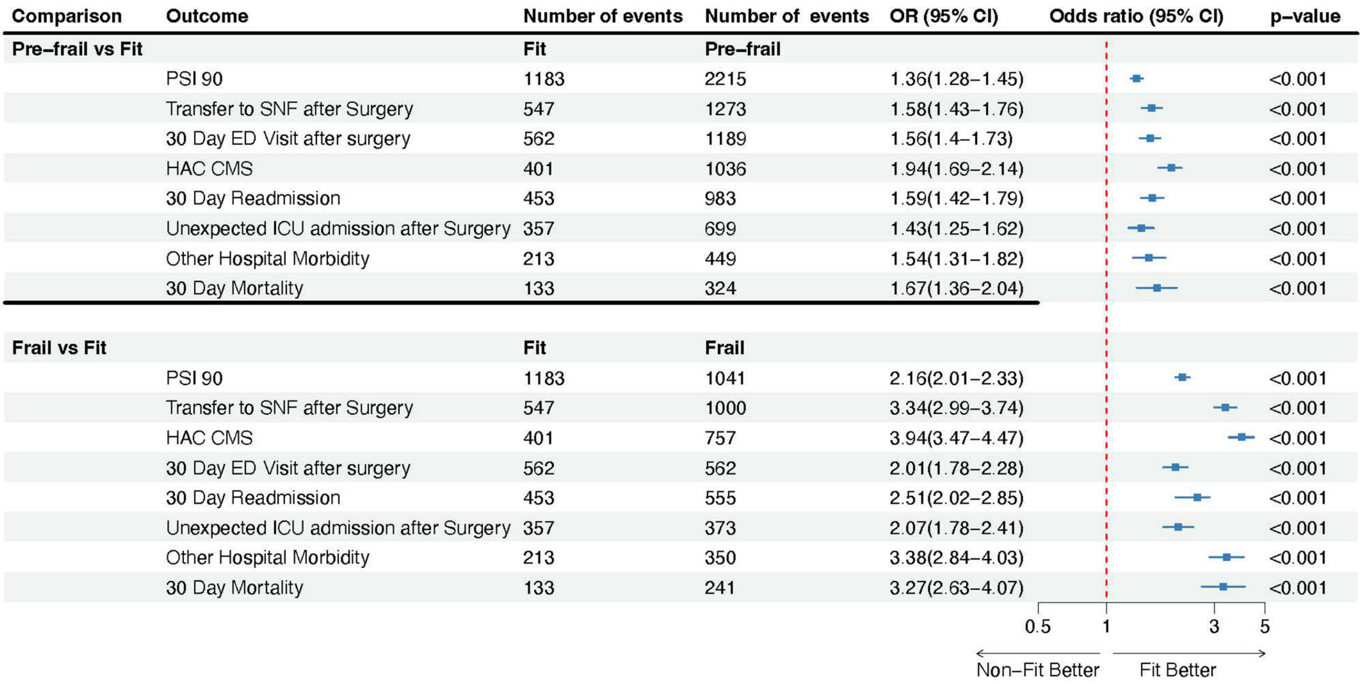

© 2023 Khanna AK et al. *JAMA Network Open*

eFigure 3: Absolute risk of the composite outcome with increasing eFI, grouped by increments of 0.03

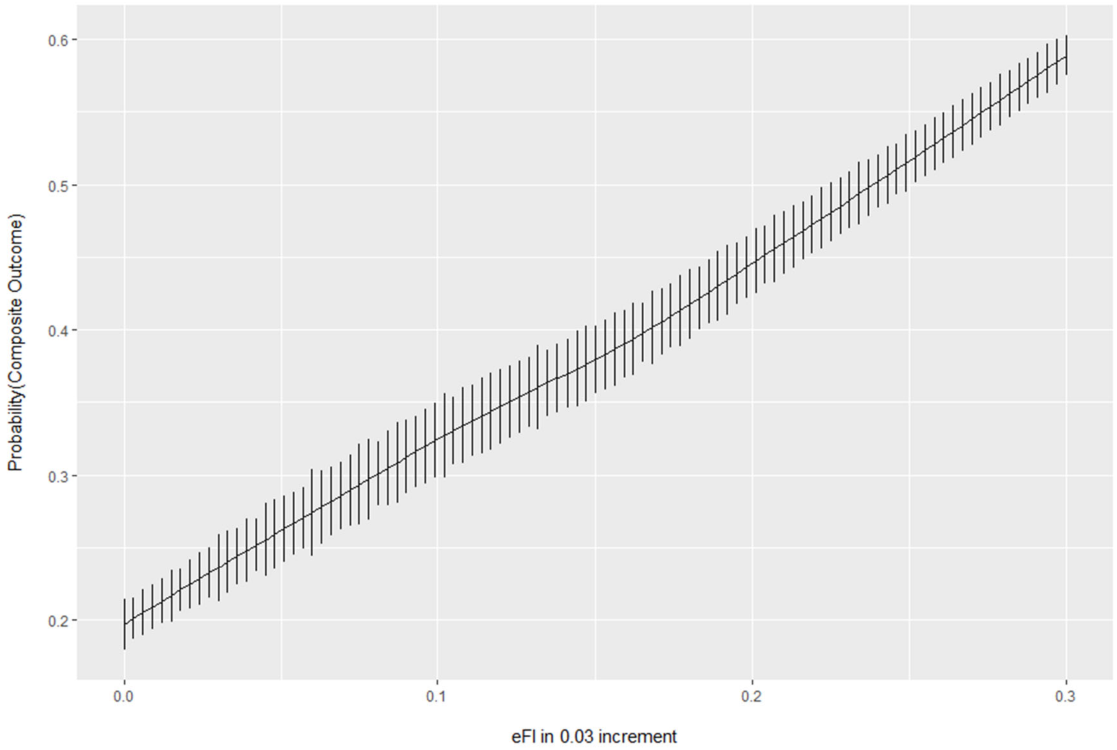

© 2023 Khanna AK et al. *JAMA Network Open*

eFigure 4: LOWESS Plot for log odds of composite outcome and eFI as a continuous variable

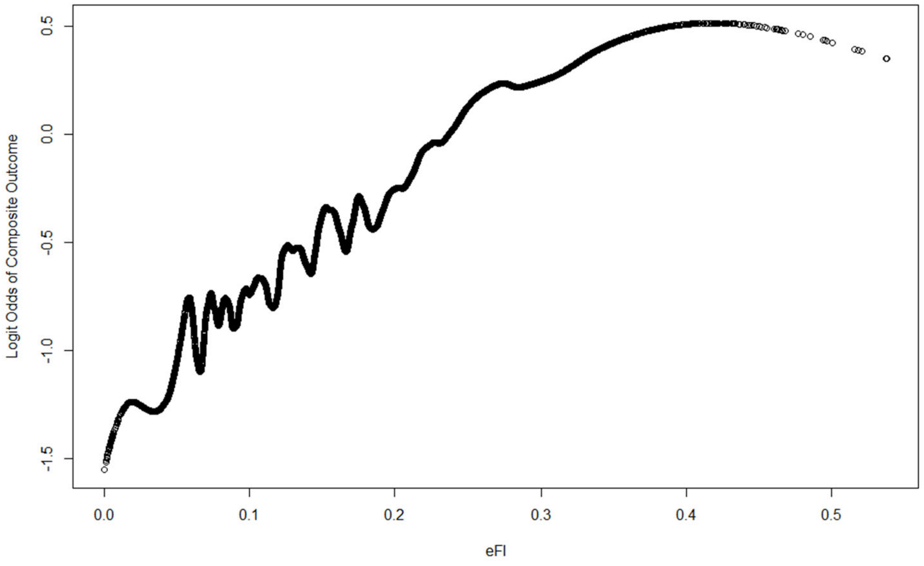

Supplement: Supplement 1. — eTable 1. The Original Construction of the Electronic Frailty Index (eFI) Spanned 54 Deficits eTable 2. Modifications to the Electronic Frailty Index (eFI) Since Its Original Publication in Pajewski et al eTable 3. Definition of Primary Composite Outcome eTable 4. Center for Medicare and Medicaid Services (CMS) Patient Safety Indicator 90 (PSI 90), Hospital-Acquired Conditions (HAC) and Other Hospital Morbidity Definitions eTable 5. Variance Inflation Factor (VIF) for Estimation of Collinearity Between Covariates eTable 6. Analysis of Excluded Patients Where eFI Was Not Calculated Since They Did Not Meet the Criteria for 2 Blood Pressure Readings in the Last Two Years eTable 7. Odds of Outcomes Across Frail Groups, Adjusted for Age, Sex and Race eTable 8. Event Rates for Study Outcomes by Electronic Frailty Index (eFI) Groups eTable 9. Hospital Acquired Conditions (HAC) per CMS Definition and Distribution in the Study Cohort eTable 10. Odds Ratios of Covariates for the Composite Outcome in a Multivariable Logistic Regression Adjusted for Significant Covariates and Charlson Comorbidity Index eFigure 1. Flow Diagram for the Study Population eFigure 2. Box Plot Showing Varying Risk of Outcomes for Electronic Frailty Index (eFI) Groups After Adjustment for Age, Gender, Race, and Charlson Comorbidity Index (CCI) eFigure 3. Absolute Risk of the Composite Outcome With Increasing eFI, Grouped by Increments of 0.03 eFigure 4. LOWESS Plot for Log Odds of Composite Outcome and eFI as a Continuous Variable [file jamanetwopen-e2341915-s001.pdf]
