# Supplementary material for: Automated Electronic Frailty Index–Identified Frailty Status and Associated Postsurgical Adverse Events
Source: JAMA Netw Open. 2023 Nov 6;6(11):e2341915. doi: 10.1001/jamanetworkopen.2023.41915 (PMC10628731; doi:10.1001/jamanetworkopen.2023.41915)
Supplement: Supplement 2. — Data Sharing Statement [file jamanetwopen-e2341915-s002.pdf]

Data Sharing Statement

Khanna. Automated Electronic Frailty Index–Identified Frailty Status and Associated Postsurgical Adverse Events. *JAMA Netw Open*. Published November 06, 2023. doi:10.1001/jamanetworkopen.2023.41915

Data
Data available: No

Additional Information
Explanation for why data not available: There are significant issues with protected health information that preclude us from providing this data.
